# Digital Technology Tools to Examine Patient Adherence to a Prescription-Only Omega-3 Polyunsaturated Fatty Acid Therapy To Mitigate Cardiovascular Risk: Protocol for a Prospective Observational Study and Preliminary Demographic Analysis

**DOI:** 10.2196/29061

**Published:** 2021-08-30

**Authors:** Gregory P Arutyunov, Alexander G Arutyunov, Fail T Ageev, Tatiana V Fofanova

**Affiliations:** 1 Pirogov Russian National Research Medical University Moscow Russian Federation; 2 National Medical Research Center for Cardiology Moscow Russian Federation

**Keywords:** omega-3-acid ethyl esters, myocardial infarction, hypertriglyceridemia, adherence, compliance, persistence, mHealth, eHealth, patient-reported outcomes

## Abstract

**Background:**

Sustained adherence and persistence with prescription medications is considered essential to achieve maximal treatment benefit for patients with major chronic, noncommunicable diseases such as hyperlipidemia and lipid-associated cardiovascular disease. It is widely documented, however, that many patients with these conditions have poor long-term adherence to their treatments. The population of Russia is affected by poor adherence in the same ways as populations elsewhere and continues to have high rates of cardiovascular disease.

**Objective:**

The purpose of this study was to examine patient adherence to a prescription-only preparation of highly purified omega-3 polyunsaturated fatty acids (1.2 to 1 eicosapentaenoic acid to docosahexaenoic ratio, 90% purity) in a large sample of patients at risk for cardiovascular diseases using digital technology to monitor patient behavior and as an outreach facility for patient education and engagement.

**Methods:**

We conducted a 6-month prospective observational study (DIAPAsOn) at >100 centers in the Russian Federation. A bespoke electronic data capture and patient engagement system were developed with a well-established Russian technology supplier that enables information obtained during clinic visits to be supplemented by remote patient self-reporting. Other aspects of the program included raising patients' awareness about their condition via educational materials available in personal patient accounts in the electronic system.

**Results:**

From an initial cohort of 3000 patients, a safety population of 2572 patients (age: mean 60 years) with an equal proportion of men and women has been characterized. There was widespread concomitant cardiovascular pathology and commensurate use of multiple classes of cardiovascular medication, notably lipid-modifying and antihypertensive drugs. The program was completed by 1975 patients, of whom 780 were prescribed highly purified omega-3 polyunsaturated fatty acid supplements for secondary prevention after myocardial infarction and 1195 were prescribed highly purified omega-3 polyunsaturated fatty acid supplements for hypertriglyceridemia. Data collection and analysis have been completed.

**Conclusions:**

DIAPAsOn will provide insights into patient adherence with prescription-grade omega-3 polyunsaturated fatty acid therapy and perspectives on the role of mobile technology in monitoring and encouraging adherence to therapy.

## Introduction

Low-density lipoprotein cholesterol (LDL-C), the primary lipoprotein component of total cholesterol, is recognized as the most important lipid risk factor for coronary heart disease [1]. An extensive array of clinical trials has demonstrated the value of lowering LDL-C as a means of prevention (especially secondary prevention) of major cardiovascular events, including myocardial infarction and stroke; meta-analyses [2,3] exemplify the influence of LDL-C levels on cardiovascular risk—every 1 mmol/L (39 mg/dL) reduction in LDL-C leads to a decrease in overall mortality of 12%, a 19% reduction in coronary mortality, and a 17% reduction in the incidence of stroke, with the effect size relating closely to the absolute reduction in LDL-C achieved and evident across the continuum of LDL-C levels. These findings underpin the status of statins as first-line therapies to reduce cardiovascular risk via modulation of LDL-C levels.

Nevertheless, in a meta-analysis [2], 5-year event rates in statin-treated patients were 14% (compared with 18% in the reference group), and even higher residual risk was apparent in patients with preexisting coronary heart disease or type 2 diabetes. It has been estimated that some 50% of individuals with acute coronary syndrome do not have elevated LDL-C, indicating that other factors account for a sizeable proportion of total cardiovascular risk [4].

The existence of residual risk in patients whose LDL-C is well controlled with medication [5,6] has focused attention on additional contributors to that persisting risk, including elevated plasma triglyceride levels [7]. Demonstration of a causal link between elevated triglycerides and cardiovascular risk has been a matter of controversy, with evidence of an association between triglyceride levels and cardiovascular risk often being attenuated after adjustment for other factors [8-12]. Considerations such as the reciprocal relationship between levels of triglyceride and levels of high-density lipoprotein cholesterol (HDL-C), which is a correlation that persists even when triglyceride are low [13], as well as appreciation of qualitative metabolic interplay between triglyceride-rich lipoproteins and HDL-C fractions, have nevertheless established a strong prima facie case for triglyceride levels as one factor influencing residual cardiovascular risk in statin-treated patients, especially in patients characterized by a high triglyceride to HDL-C ratio. Various national and international guidelines recognize that elevated triglyceride may be implicated in the overall risk of coronary heart disease [14-16], notably so in patients with type 2 diabetes, obesity, or metabolic syndrome [17-20].

Highly purified long-chain omega-3 polyunsaturated fatty acids (n-3 PUFAs), available as prescription-only medications, are approved in various countries for the management of elevated triglyceride. These preparations are qualitatively distinct from dietary supplements of n-3 PUFAs [21]. One such prescription n-3 polyunsaturated fat preparation (OMACOR, Market Authorization Holder Abbott Laboratories GmbH), is available in Russia as 1 g capsules containing 840 mg of ethyl esters of omega-3 fatty acids—a 1.2 to 1 ratio (eicosapentaenoic acid to docosahexanoic acid, 90% purity)—and 160 mg of excipients. This preparation (hereafter designated OM3EE) is approved for doses of 2 to 4 g per day, for the regulation of triglyceride levels, and at a daily dose of 1 g, for the secondary prevention of major cardiovascular events in patients after myocardial infarction; the latter indication was supported by the findings of a randomized clinical trial [22] and corroborated by a recent Cochrane review [23].

Continued therapy is central to the attainment of the full and sustained benefit in cardiovascular prevention and a range of other major noncommunicable diseases. Patients’ ownership of their situation, and the accompanying empowerment, can be an important determinant of willingness to persevere with a course of therapy [24]. This can be a particular challenge when dealing with initially symptomless conditions such as hyperlipidemia, where the connection between aberrations in blood lipid levels and later major cardiovascular events can seem abstract.

Previous trials [22,25] have highlighted the importance of long-term adherence to omega-3–based therapy in cardiovascular preventive medicine. The highly purified n-3 PUFAs are generally considered to be well tolerated and largely free of substantial adverse effects but even so a sufficient degree of patient adherence to therapy outside the framework of a controlled clinical trial cannot be taken for granted. This consideration—combined with the evidence from GISSI-Prevenzione [22] and the JELIS study [25] that good adherence maintained over a course of years can deliver clear clinical benefits—makes better understanding of treatment adherence in real-world circumstances desirable.

The emergence of widely available digital and internet technologies with the potential to provide rapid or immediate bidirectional communication between health care professionals and patients may be an important new resource for promoting long-term adherence to therapies [26]. DIAPAsOn was designed to study patient adherence to OM3EE therapy using digital technology tools.

## Methods

### Overview

DIAPAsOn was a nonrandomized prospective study (Clinicaltrials.gov NCT03415152) conducted at >100 centers in the Russian Federation to assess adherence to OM3EE prescribed as either a secondary preventive medical therapy for patients with a history of recent myocardial infarction (approved dose 1 g per day) or for lipid level regulation in patients with endogenous hypertriglyceridemia who have had insufficient responsiveness to either dietary modification or other drug therapy (approved dose 2-4 g per day). Because the objectives of the program are exploratory, there was no formal study hypothesis and no formal calculation of sample size; however, we planned to enroll 3000 patients.

There was no control group in DIAPAsOn and patients did not undergo diagnostic tests or interventions other than those that are currently accepted standards of medical care.

We used digital technology to facilitate patient-initiated data collection. Patients accessed a customized electronic system that allowed them to enter data, including daily records of OM3EE administration, and to complete various questionnaires relating to, for example, health-related quality of life and product usability on a 4-level scale (very good, good, moderate, poor). If the reported grade was moderate or poor, patients were asked to provide narrative details. This system included an option for patients to set up reminders to take the study medication. Patients were also able to report side effects in their personal accounts or to indicate if they had undergone any cardiovascular-related, new angina pectoris, or nonfatal myocardial infarction hospitalizations.

### Recruitment of Study Centers and Patients

Selection of investigators and sites to participate in the program was based on the ability to properly conduct the program, including capacity to complete an electronic case record form, and existence within the study center of a cohort of adult (≥18 years) patients with a history of myocardial infarction not earlier than 6 months for whom OM3EE was prescribed as part of a medical secondary prevention regimen or existence within the study center of a cohort of patients with a diagnosis of hypertriglyceridemia not adequately controlled by a hypolipidemic diet.

Patients satisfying these criteria at the selected sites were eligible to participate in DIAPAsOn if they had been taking OM3EE for no more than 14 days at the time of enrollment and if they were considered capable, either personally or with the assistance of immediate relatives, of submitting data through a mobile phone app or web browser. The use of this system was voluntary, and once enrolled, patients remained part of the study whether or not they used the system or withdrew during the observation period. No a priori assumption was made regarding any possible changes in treatment or follow-up related to the use of the system.

Candidates for enrollment were excluded if they were taking other prescription-only n-3 PUFAs or nutrition supplements containing omega-3 polyunsaturated fats at screening or had taken such compounds within the previous 6 months. Other exclusion criteria were being pregnant or breastfeeding; having a known sensitivity to the active substance, excipients, or soy; having been diagnosed with exogenous hypertriglyceridemia (Frederickson type I hyperchylomicronemia); participating in any other clinical or observational study at the same time as DIAPAsOn or within the previous 30 days; or having any other clinical state that, in the opinion of the center investigator, made the patient unsuitable for inclusion.

### Schedule of Visits and Data Collection

**Figure 1:**
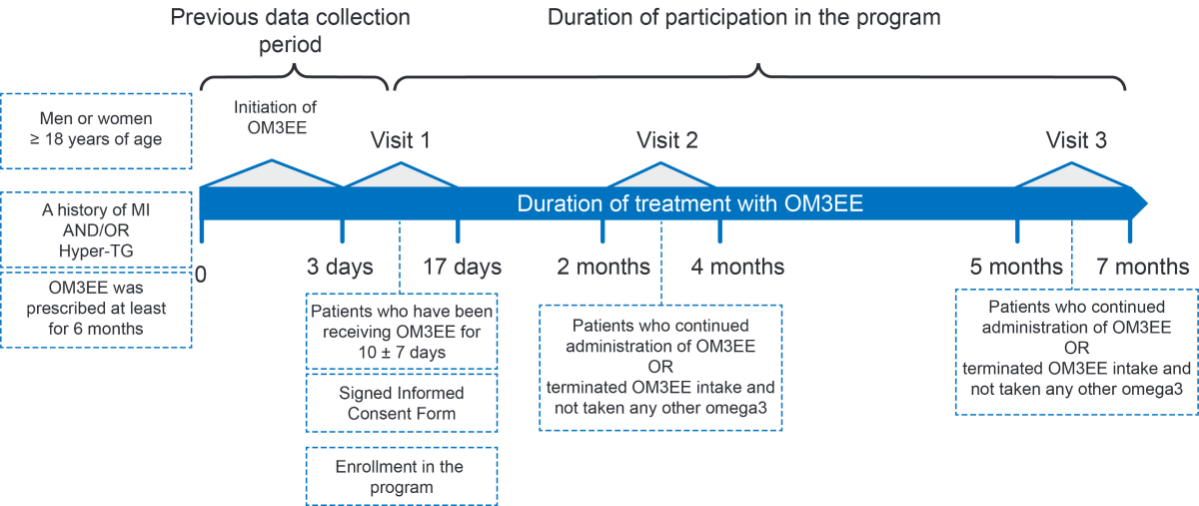
Design and chronology of the DIAPAsOn study. MI: myocardial infarction; TG: triglyceride.

DIAPAsOn included 3 principal clinic visits (Figure 1). At each visit, patients were asked about their adherence to OM3EE using the Questionnaire of Treatment Adherence [27]. This instrument, which has been used to investigate adherence to antihypertensive medication among Russian patients, produces a numerical indication of adherence: 12 to 15 points is very high, 8 to 11 points is high; 4 to 7 points is moderate, and 0 to 3 points is low. Concomitant diseases at the first visit were classified according to International Classification of Diseases, tenth revision (ICD-10) codes.

At these visits, information about concomitant therapies, blood pressure, and heart rate were also obtained. Because OM3EE was administered within the frames of normal medical practice rather than being supplied by the sponsor, information about the batch number and shelf life of the supplement were recorded at each visit.

Blood lipid profile was determined at each study visit. No central laboratory procedures were performed. All laboratory tests were conducted in accordance with routine local clinical practice.

Safety and adverse events data were recorded at each visit. In the event of a patient discontinuing the study, enquiries were made about the reason (adverse drug reaction; lack of effect; inconvenient to use; not available in pharmacies; other).

The principal clinic visits were supplemented by monthly investigator-led phone calls that focused on adherence to therapy and safety. The investigator asked the patient for information about medication batch number and expiry date if this information was not collected during regular physician visits.

### Mobile Patient Engagement Technology

The electronic patient engagement and data collection system used in DIAPAsOn was developed in collaboration with the medical online platform ROSMED [28], which has extensive experience in the development and operation of such facilities in the Russian Federation, including the Russian Severe Asthma Registry (RSAR NCT03608566), the all-Russian register of patients with hearing impairment and the integrated support program (anticipated recruitment 12 million people), and the Russian Register of patients aged >80 years with acute coronary syndrome, and maintenance, on behalf of the Russian Glaucoma Society, of the first Russian pharmaco-epidemiological study of the treatment of glaucoma patients with retinoprotective interventions and adherence to therapy [28]. The platform satisfies current requirements of Russian legislation pertaining to the management of personal data and the implementation of observational studies, primarily Russian Federal Law 152, and is included in the Russian Unified Register of computer programs and databases of the Ministry of Communications of Russia; certification of quality management and information security management has been conducted in accordance with ISO 9001:2015 and ISO/IEC 27001:2013 [28].

The system devised for DIAPAsOn was configured to minimize technical and ergonomic barriers to participant adoption and use. Contributing patients could work with the web version (configured to work with all popular browsers) or download the smartphone app (Apple iOS or Android).

Obligatory form fields for remote completion by patients included (1) medication consumption on a daily basis; (2) assessment of quality of life using the SF-36 questionnaire [29] (every 3 months); (3) evaluation of medication usability (month 1); and (4) reason for termination of the treatment, specifying adverse effects, lack of effect, inconvenience of use, nonavailability, or other. Optional form fields included (1) adverse effects (if “Yes,” the electronic system sent a notification message to the attending physician and the patient received the message, “Your doctor received the message about changes of your health”); (2) hospitalizations for cardio-vascular reasons, new cases of stenocardia, or nonfatal myocardial infarction; and (3) tests of patient’s knowledge of the problem or disease based on acquaintance with study materials available in the electronic system of the program.

**Figure 2:**
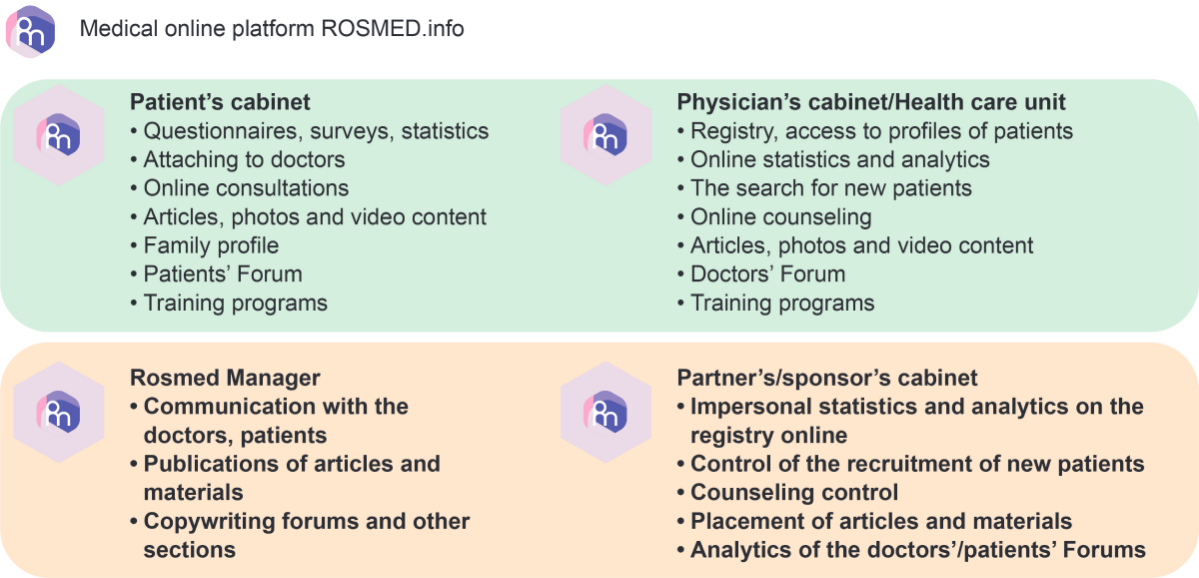
User interactions.

**Figure 3:**
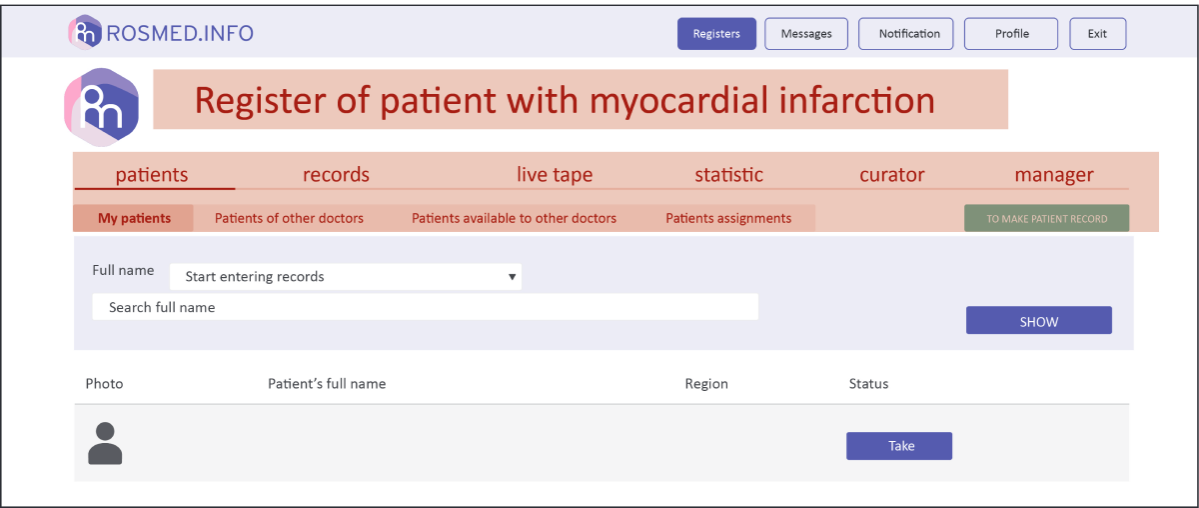
Physician’s cabinet graphical user interface.

Operational domains of the digital platform (Figure 2) consist of an administrative tier, occupied by the service manager and the study sponsor, and an executive tier, which includes dedicated areas (cabinets) for use by patients and physicians (Figure 3; these screenshots have been translated into English, and the examples shown here have also been anonymized).

**Figure 4:**
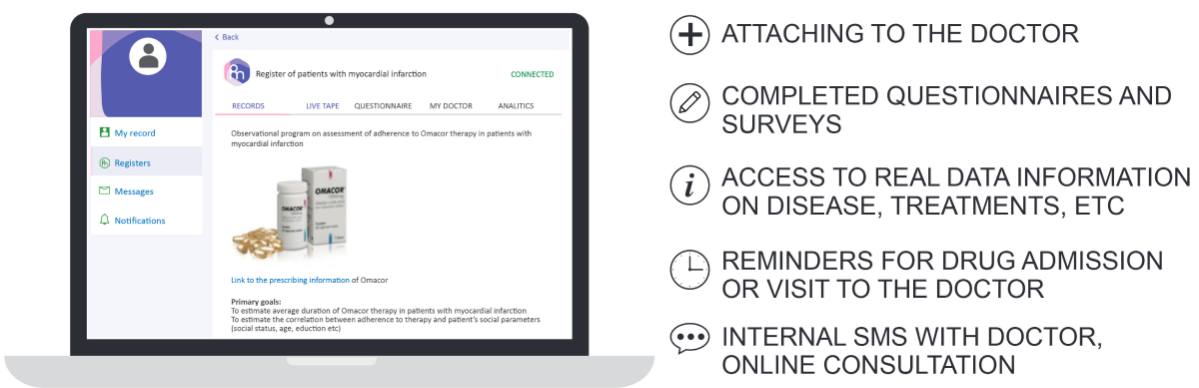
Patient’s cabinet.

**Figure 5:**
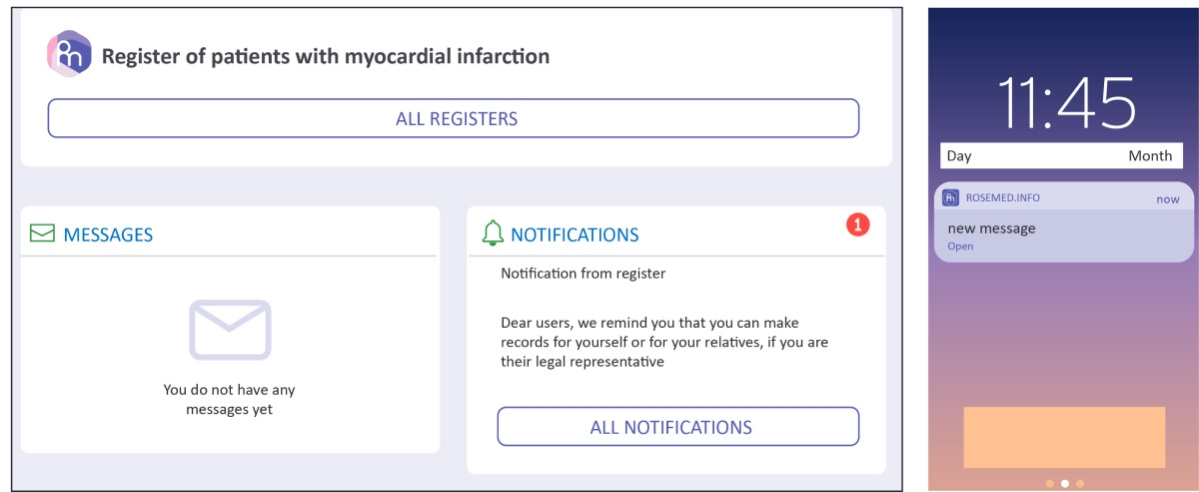
Notices and push-reminder messages.

**Figure 6:**
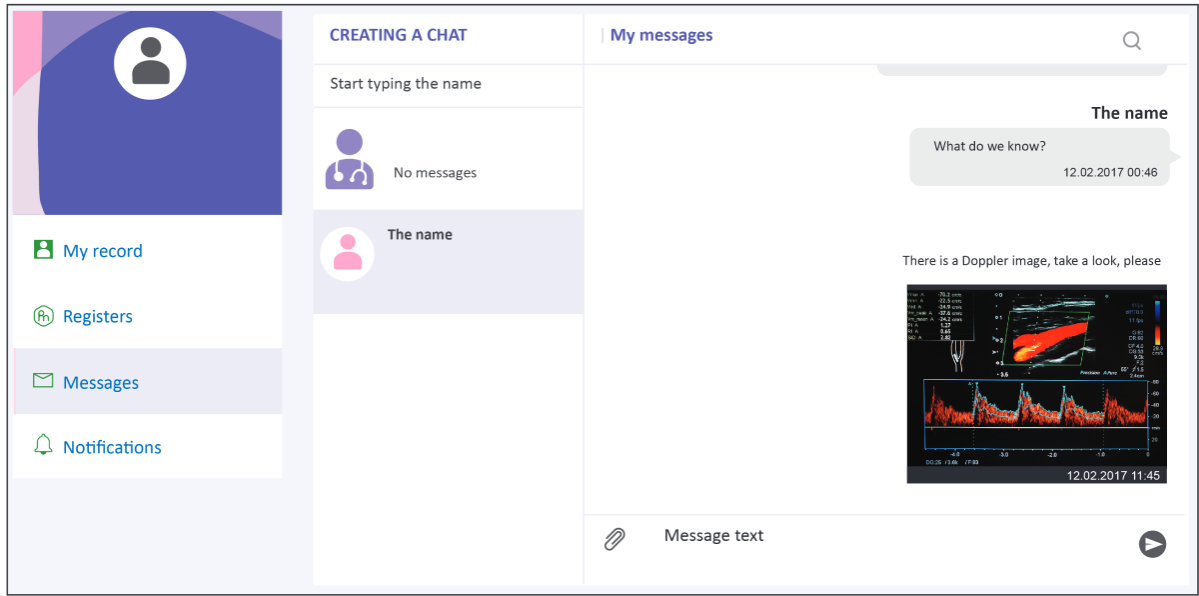
Internal social network.

**Figure 7:**
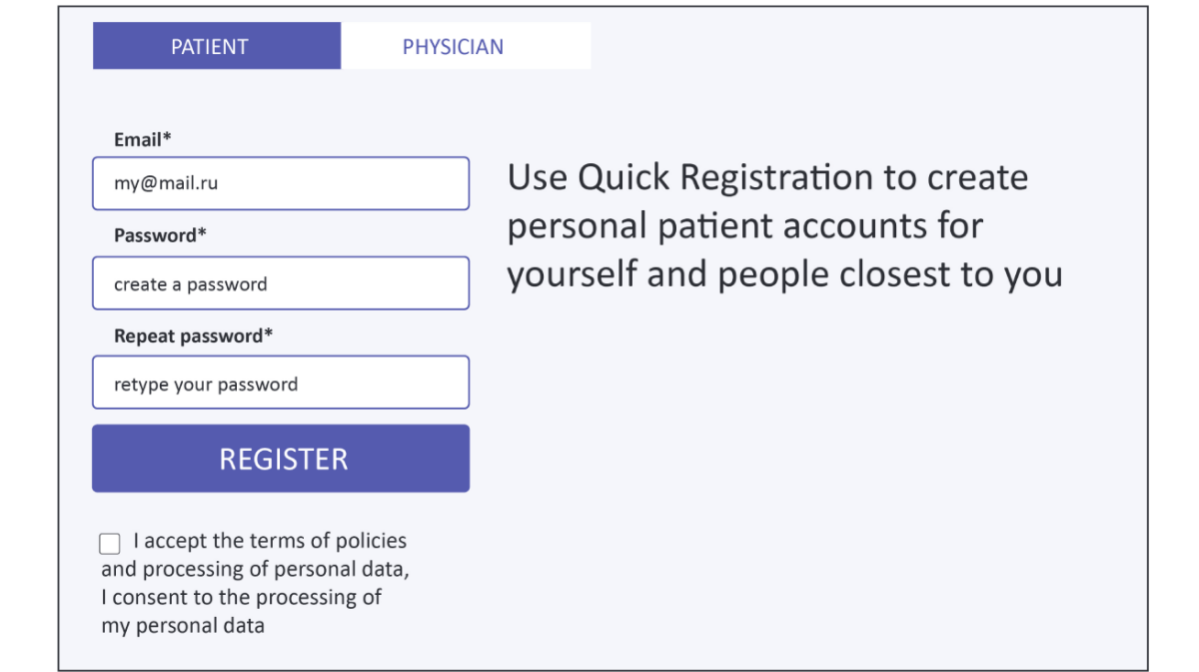
Simplified patient registration.

The landing page for the Patient Cabinet (Figure 4) displays 5 drop-down menus designed to allow patients easy access to the full range of services. These include access to notices and push-reminder services (Figure 5) and an internal social network (Figure 6). The patient registration page was deliberately kept simple to minimize set-up friction and encourage patient enrollment (Figure 7).

### Statistical and Analytical Plans

Statistical analysis was performed in accordance with a preapproved Statistical Analysis Plan, using R (version 3.4.3; The R Project).

We used the 2-tailed paired *t* test for dependent samples for continuous data and the McNemar test for categorical data for comparisons between scheduled visits. For all comparisons, statistical significance was calculated at the threshold of *P*<.05.

For applicable endpoints, further analysis was performed in subgroups of patients with different adherence rates (ranked on a 0-1 scale) at visits 2 and 3.

The primary endpoint was assessed per-protocol (all patients for whom data were collected), at least at visit 2. The safety population included all patients who completed at least visit 1. This population was used to record reports of adverse drug reactions, serious adverse drug reactions, and other safety information.

Analysis of the primary endpoint included (1) determining the mean adherence rate, calculated as the sum of days when the patient took the full prescribed dose of highly purified omega-3 polyunsaturated fatty acid supplement during the specified period divided by the total number of days in that period, at the end of the study (visit 3); and (2) mean score on the National Questionnaire of Treatment Compliance, determined at the end of the study (visit 3).

### Ethics

Ethical oversight of the study was exercised by the independent Interuniversity Ethics Committee. Initial written approval was issued on October 19, 2017, before the start of the study. The study was then implemented in accordance with the protocol version of November 8, 2017.

The study conformed to the requirements of Good Clinical Practice and to all applicable national standards relevant to the rights, safety, and well-being of all study participants in accordance with the provisions of the Declaration of Helsinki and all relevant national legislation and related provisions. Informed written consent was obtained from the patient to use and disclose personal and medical information. Prospective patients were apprised of their right to decline further participation in the study at any time and for any declared reason (or for no reason) without prejudice to any subsequent treatment.

Individual center investigators were responsible for ensuring quality control; execution of the program; and the collection, documentation, and submission of data in accordance with the protocol, standards of Good Clinical Practice, and all applicable local laws.

### Administrative Structure

**Figure 8:**
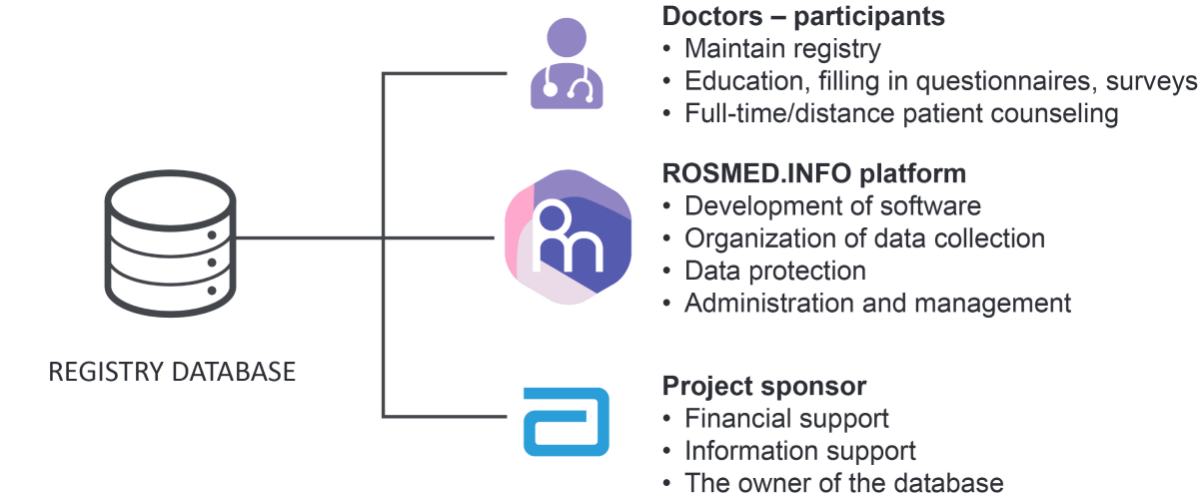
Functional scheme of the project.

The tripartite functional organization of the project is illustrated in Figure 8. A list of 107 investigators at participating centers is presented in Multimedia Appendix 1.

Abbott Laboratories (Moscow), in its role as study sponsor led the development of the protocol, subject to endorsement by the investigators, and discharged statutory responsibilities with respect to drug safety and adverse events monitoring. Abbott Laboratories (Moscow) did not supply highly purified omega-3 polyunsaturated fatty acid supplement, which was prescribed in the context of routine clinical practice. Results were collated and analyzed by an independent external biostatistician. Financial compensation for physicians was reasonable and represented local fair market value for the services provided. Patients in the study did not receive any financial compensation.

## Results

A total of 3000 patients were initially included in the program, but 428 (14.3%) were excluded because visit 1 data were incomplete. Valid and complete data from visit 1 were available for 2572 of the 3000 patients (85.7%), who constituted the safety population (Table 1), and data from a per-protocol contingent of 2167 patients were accrued (Table 2); 405 patients failed to complete at least visit 2.

Within the per-protocol population, 41.4% (898/2167) were prescribed OM3EE for secondary prevention after myocardial infarction, and 58.6% (1269/2167) for hypertriglyceridemia. The program was completed by 1975 patients, 780 of whom were being treated for secondary prevention, and 1195 of whom were being treated for hypertriglyceridemia. Our protocol stipulated that discontinuation of the study medication was not a reason to withdraw a patient from the study.

Men accounted for 52.8% (1145/2167) of the total population and 67.7% (608/898) of the subgroup prescribed OM3EE for secondary prevention postmyocardial infarction; conversely, most of the patients prescribed OM3EE for hypertriglyceridemia were women (732/1269, 57.7%). The study population was almost exclusively Caucasian (2118/2167, 97.7%), and almost all other patients (43/2167, 2.0%) were Asian.

Patients’ mean age was 59.9 years (SD 11.6), and overall mean BMI was 29.9 kg/m^2^ (SD 5.6). Slightly more than one-quarter of all patients (613/2167, 28.3%) smoked actively. In the secondary prevention subgroup, the mean time since the index myocardial infarction to the start of study therapy with highly purified omega-3 polyunsaturated fatty acid supplement was 3.04 months (SD 1.7).

The most frequently recorded concomitant diseases were hypertensive diseases (ICD-10 codes: I10-I15), recorded in 63.8% (1383/2167) of patients. Within those categories, most patients were diagnosed with hypertensive heart disease without congestive heart failure (725/2167, 33.5%) and essential hypertension (328/2167, 15.1%). Coronary heart disease (ICD-10 codes: I20-I25) was recorded in 17.9% of patients (388/2167), other heart disease (ICD-10 codes: I30-I52), including unspecified atrial fibrillation and atrial flutter, was recorded in 16.2% (352/2167) and diabetes was recorded in 7.1% of patients (154/2167). Metabolic disorders (ICD-10 codes: E70-E90) were recorded in 10.8% of patients (235/2167).

Therapies prescribed during the 12 months preceding enrollment were mostly lipid-modifying agents (1600/2167, 73.8%). The single most frequently prescribed drug in this category was rosuvastatin (906/2167, 41.8%), followed by atorvastatin (601/2167, 27.7%). Combinations of lipid-modifying drugs accounted for 6.9% (150/2167) of prescriptions. Over half of the patients (1140/2167, 52.6%) had been prescribed antiplatelet agents, primarily aspirin (n=583), and 40.6% (879/2167) had been prescribed beta-blockers, predominantly bisoprolol (n=586). In addition, 694 patients (694/2167, 32%) received angiotensin-converting enzyme inhibitors, either as monotherapy (n=438) or in combination with other classes of antihypertensive drugs, including diuretics and calcium-channel blockers. Perindopril was the most widely prescribed angiotensin-converting enzyme inhibitor (n=325).

Variations were apparent in the 2 indication-specific subgroups. The use of anticoagulants was recorded in 96.4% (866/898) of patients who received OM3EE for secondary prevention postmyocardial infarction. The use of beta-blockers was recorded in 62.9% (564/898), the use of angiotensin-converting enzyme inhibitors was recorded in 31.9% (286/898), and the use of lipid-modifying agents was recorded in 73.6% (661/898). Prestudy use of lipid-modifying agents was at a similar level in patients with hypertriglyceridemia (939/1269, 74.0%) but the use of above-specified classes of agents was at a lower level.

Baseline mean systolic blood pressure was 133.7 mmHg (SD 17.3) and baseline mean diastolic blood pressure was 81.4 mmHg (SD 8.8). Some patients, 21.4% in the overall cohort (463/2167) and 23.6% in the hypertriglyceridemia subset (299/1269), but only 18.0% of those in the postmyocardial infarction subgroup were considered to have clinically relevant elevation of systolic blood pressure. Approximately 13% (hypertriglyceridemia: 165/1269; postmyocardial infarction: 117/898) were considered to have clinically relevant elevation of diastolic blood pressure.

**Table 1:** Safety population characteristics (participants with data available from visit 1).

Characteristic	Overall population (n=2572), mean (SD)	Secondary prevention postmyocardial infarction (n=1171), mean (SD)	Hypertriglyceridemia (n=1401), mean (SD)
Age (years)	59.9 (11.6)	61.8 (10.3)	58.2 (12.4)
Height (m)	1.7 (0.1)	1.7 (0.1)	1.7 (0.1)
Weight (kg)	87.1 (16.9)	86.0 (15.1)	88.0 (18.1)
BMI^a^ (kg/m^2^)	29.9 (5.6)	28.5 (4.1)	31.0 (6.4)

^a^BMI: body mass index.

**Table 2:** Per-protocol population sociodemographic characteristics.

Characteristic			Per protocol (n=2167), n (%)		Secondary prevention postmyocardial infarction (n=898), n (%)		Hypertriglyceridemia (n=1269), n (%)
**Sex**							
	Male	1145 (52.8)		608 (67.7)		537 (42.3)	
	Female	1022 (47.2)		290 (32.3)		732 (57.7)	
**Education**							
	Some secondary	17 (0.8)		13 (1.4)		4 (0.3)	
	Secondary general	238 (11.0)		86 (9.6)		152 (12.0)	
	Secondary vocational	673 (31.1)		280 (31.2)		393 (31.0)	
	Higher	1199 (55.3)		504 (56.1)		695 (54.8)	
	Supplementary vocational	40 (1.8)		15 (1.7)		25 (2.0)	
**Work status**							
	Working	1199 (55.3)		445 (49.6)		754 (59.4)	
	Not working	968 (44.7)		453 (50.4)		515 (40.6)	
**Marital status**							
	Single	91 (4.2)		33 (3.7)		58 (4.6)	
	Married	1744 (80.5)		669 (74.5)		1075 (84.7)	
	Divorced	124 (5.7)		60 (6.7)		64 (5.0)	
	Widowed	208 (9.6)		136 (15.1)		72 (5.7)	
**Smoking status**							
	Smokes	613 (28.3)		284 (31.6)		329 (25.9)	
	Does not smoke	1554 (71.7)		614 (68.4)		940 (74.1)	

## Discussion

Rates of death from coronary heart disease have been falling in Russia in recent decades [30], but the rate of decline has been less marked than in other European countries, and age-standardized mortality rates remain markedly higher than those in other nations [31]. Data from other investigations suggest that not all of this discrepancy in declines can be explained by blood lipid levels (alcohol appears to exert a notable influence [32]), but lipid levels are regarded as central determinants of risk [33]. Additional efforts to improve the management of coronary risk are, therefore, warranted; a focus on patient adherence to secure maximum benefit from available therapies is one logical dimension of that response.

It is clear from CEPHEUS II data that failure to reach targets for lipid-based risk reduction is widespread in Russia [34]. Patient-related factors associated with nonattainment of targets identified in that study included patients considering it acceptable to miss prescribed doses more than once per week.

In response to these deficits, we developed digital technology tools designed to stimulate patient engagement and education and recruited 3000 patients to study the effectiveness of this mobile health technology. We believe this research in preventive cardiovascular medicine to be the first of its kind undertaken at scale in Russia: it may provide a model for further studies in this field.

The patient population of DIAPAsOn was large and was recruited from a wide geographical area. This gives us the confidence that the demographic profile and extensive use of concomitant cardiovascular medications reported herein are likely an accurate reflection of the patient population in the Russian Federation that are likely to be candidates for prescription-grade n-3 PUFA therapy. As such, we are optimistic that the findings of DIAPAsOn may be instructive about the routine use of prescription-grade n-3 PUFA therapy throughout the Federation, which has a population of 145 million.

The limitations of this study must be recognized. The absence of a control group precludes any determination of cause and effect, and the potential for biases in any trial of this type must be acknowledged. A retrospective calculation of the Nichol score [35] for DIAPAsOn confirmed that our study rated favorably in the *Disease-Related Criteria* and *Compliance Definition and Measurement Criteria* subcategories but scored less strongly in the *Study Design Criteria* subcategory. The duration of follow-up was appropriate for an initial evaluation of a technical innovation in the management of chronic cardiovascular risk [36], but a substantially longer period of observation (we conjecture at least 1 year) will be needed to demonstrate whether our strategy for digital patient engagement translates into a robust, sustained, and meaningful improvement in long-term adherence. The web-based resources were developed and presented in Russian, and the study population was highly homogeneous: those factors may restrict the extrapolation of findings from this study to other countries. We did not perform a prestudy evaluation of the web-based services used in DIAPAsOn using, for example, the Mobile App Rating Scale, or a prefatory feasibility study, such as that reported by Paldán et al [37].

These reservations notwithstanding, experience and insight from this prospective study of a digital technology and patient self-reporting platform to enhance adherence may inform further development of mobile health engagement technologies for Russian populations to improve adherence to medications and thereby reduce the risk of clinical cardiovascular events.

## Appendix

Multimedia Appendix 1 DIAPAsOn center investigators.

## Abbreviations

BMI: body mass index
HDL-C: high-density lipoprotein cholesterol
ICD-10: International Classification of Diseases, tenth revision
LDL-C: low-density lipoprotein cholesterol
n-3 PUFA: omega-3 polyunsaturated fatty acid
