# Supplementary material for: Digital Technology Tools to Examine Patient Adherence to a Prescription-Only Omega-3 Polyunsaturated Fatty Acid Therapy To Mitigate Cardiovascular Risk: Protocol for a Prospective Observational Study and Preliminary Demographic Analysis
Source: JMIR Res Protoc. 2021 Aug 30;10(8):e29061. doi: 10.2196/29061 (PMC8438613; doi:10.2196/29061)
Supplement: Multimedia Appendix 1 [file resprot_v10i8e29061_app1.pdf]

Region	Investigator
Astrakhan Oblast	1. Borodulya Mihail Vasil'evich
Bryansk Oblast	2. Petuhova Irina Leonidovna
Vladimir Oblast	3. Timofeeva Irina Vladimirovna
Volgograd Oblast	4. Vorob'eva Svetlana Gennad'evna
	5. Gulova Ol'ga Aleksandrovna
	6. Dugencova Larisa Anatol'evna
	7. Sytilina Natal'ya Nikolaevna
Voronezh Oblast	8. Trubicyna Irina Valentinovna
Kemerovo Oblast	9. Krestova Ol'ga Sergeevna
Krasnodar Krai	10. Alekseeva Elena Valer'evna
	11. Allaabed Hassan Adnanovich
	12. Bahmet'eva Irina Aleksandrovna
	13. Boldin Vasilij Borisovich
	14. Vikulova Larisa Vladimirovna
	15. Golovko Artyom Yur'evich
	16. Emuzova Liana Anatol'evna
	17. Zaharov Aleksandr Yur'evich
	18. Ivochkina Marina Ivanovna
	19. Imamutdinova Marina Vladimirovna
	20. Kovalenko Fyodor Andreevich
	21. Minasyan Ani Kamoevna
	22. Mkrtycheva Angelina Gennad'evna
	23. Nikitina Elena Valer'evna
	24. Novikova Svetlana Valer'evna
	25. Pavlovec Vadim Petrovich

Region	Investigator	
	26.	Perkova Elena Mihajlovna
	27.	Prozorovskaya Yuliya Igorevna
	28.	Ramenskaya Tat'yana Evgen'evna
	29.	Raff Stanislav Anatol'evich
	30.	Smolina Elena Garievna
	31.	Subbotina Anastasiya Vladimirovna
	32.	Tatarinceva Zoya Gennad'evna
	33.	Usmanova Natal'ya Aleksandrovna
	34.	Hazhbieva Milana Musaevna
	35.	Shadzhe Evgeniya Azamatovna
Krasnoyarsk Krai	36.	Hamyt- Kyzy Ajperi Hamytovna
Moscow	37.	Adamyan Margarita Mamikonovna
	38.	Belov Leonid L'vovich
	39.	Brodichko Alena Ivanovna
	40.	Bugaev Timofej Dmitrievich
	41.	Vahrulina Natal'ya Konstantinovna
	42.	Grigor'eva Ekaterina Anatol'evna
	43.	Dvorina Ol'ga Gennad'evna
	44.	Dmitrieva Irina Mihajlovna
	45.	Karaeva Aida Anzorovna
	46.	Novosel'ceva Ekaterina Pavlovna
	47.	Oganesyan Lala Konstantinovna
	48.	Polyakova Natal'ya Olegovna
	49.	Ryzhova Tat'yana Vladimirovna
	50.	Smirnova Ol'ga L'vovna
	51.	Tavleeva Svetlana Nikolaevna

Region	Investigator	
	52.	Hrulenko Svetlana Borisovna
	53.	Chernushenko Tat'yana Ivanovna
Moscow Oblast	54.	Gukov Konstantin Aleksandrovich
	55.	Gukova Tat'yana Sergeevna
	56.	Malyarenko Elena Nikolaevna
	57.	Sorokin Sergej Anatol'evich
Nizhny Novgorod Oblast	58.	Abramova Nataliya Arkad'evna
	59.	Aksenova Nataliya Aleksandrovna
	60.	Barsukova Nataliya Aleksandrovna
	61.	Budarina Yuliya Gennad'evna
	62.	Grushin Dmitrij Valer'evich
	63.	Kolesnichenko Irina Vyacheslavovna
	64.	Lokonova Larisa Mihajlovna
	65.	Fedorova Svetlana Nikolaevna
Novosibirsk Oblast	66.	Shurkevich Anastasiya Alekseevna
Omsk Oblast	67.	Minzhasarova Saniya Hasangalievna
	68.	Naumov Dmitrij Valer'evich
Perm Krai	69.	Zhuravleva Natal'ya Alekseevna
The Republic of Adygea	70.	Shekhmirzova Dzhanetta Ruslanovna
The Republic of Bashkortostan	71.	Gilyaeva El'vira Fanisovna
	72.	Dmitriev Aleksej Valer'evich
	73.	Murasova Rimma Ismagilovna
	74.	Tarzimanova Yuliya Shamilevna
	75.	Timerbulatov Timur Rasfarovich
Tatarstan	76.	Ivanova Natal'ya Mihajlovna
Rostov Oblast	77.	Budanova Ol'ga Veniaminovna

Region	Investigator
	78. Lobe Aleksandra Ovanesovna
	79. Mazruho Marina Karpovna
	80. Morgun Nina Karpovna
	81. Stupina Anna Alekseevna
Ryazan Oblast	82. Grusheckaya Irina Stanislavovna
	83. Samarceva Yana Nikolaevna
Samara Oblast	84. Aristova Tat'yana Vladimirovna
	85. Reznik Irina Mihajlovna
	86. Rybina Evgeniya Dmitrievna
	87. Sapunkova Svetlana Mihajlovna
	88. Filippovskaya Natal'ya Igorevna
	89. Chernova Viktoriya Nikolaevna
Saint Petersburg	90. Bulycheva-Samohina Lyubov' Vasil'evna
	91. Omel'chenko Marina Yur'evna
	92. Saf'yanova Natal'ya Viktorovna
Saratov Oblast	93. Mihajlova Elena Aleksandrovna
Smolensk Oblast	94. Novik Lyudmila Mihajlovna
Stavropol Krai	95. Vedeneva Elena Viktorovna
	96. Eremenko Aleksej Mihajlovich
	97. Kubanova Asiyat Borisovna
	98. Minasova Elena Nikolaevna
Tomsk Oblast	99. Zubova Ol'ga Valer'evna

Tula Oblast	100.	Barabanova Tat'yana Yur'evna
	101.	Dabizha Viktoriya Gennad'evna
	102.	Kolomejceva Tat'yana Mihajlovna
	103.	Prihod'ko Tat'yana Nikolaevna
Tumen Oblast	104.	Ahshiyatova Nastya Ibragimovna
Khabarovsk Krai	105.	Koroleva Ramilya Lotfullovna
Chelyabinsk Oblast	106.	Malyutina Anastasiya Gennad'evna
	107.	Fanina El'vira Rinatovna
